# Seroprevalence of toxocariasis among allergic patients in Kuwait and its association with eosinophilia

**DOI:** 10.1016/j.parepi.2022.e00260

**Published:** 2022-07-02

**Authors:** Mohammad Al-Awadhi, Wafaa Jamal

**Affiliations:** a Research Core Facility, Faculty of Medicine, Kuwait University, Jabriya, Kuwait; b Department of Microbiology, Faculty of Medicine, Kuwait University, Jabriya, Kuwait

**Keywords:** Toxocara, Toxocariasis, Nematode, Seroprevalence, Eosinophilia

## Abstract

Toxocariasis is a worldwide helminthic infection which is transmitted from infected dogs and cats and has been associated with peripheral blood eosinophilia. The Centers for Disease Control and Prevention (CDC) placed toxocariasis among the top 6 parasitic diseases in the USA which are prioritized for public health action. To our knowledge, there are no reports on human toxocariasis in Kuwait or in the other Gulf Cooperation Council (GCC) countries. This study aims at investigating the seroprevalence of toxocariasis among allergic patients in Kuwait and its association with eosinophilia, age, gender, nationality, and history of direct contact with dogs or cats. From September to December 2021, the laboratory records of allergic patients referred to Al-Rashed Allergy Hospital, Kuwait were reviewed and a total of 400 serum samples were selected: 200 samples from patients with normal eosinophil count (< 500 cells/μl) and 200 samples from patients with eosinophilia (> 500 cells/μl). The sera were screened for anti-*Toxocara canis* IgG antibodies via antibody enzyme-linked immunosorbent assay (Ab-ELISA). The seropositive patients were asked about their history of direct contact with dogs or cats. Statistical analyses were performed using Microsoft Excel® Analysis ToolPak software. Toxocariasis seropositivity was detected in 10 out of 400 (2.5%) allergic patients. Five patients had eosinophilia while 5 had normal eosinophil count. There was no difference in mean age or gender between *Toxocara*-seropositive and seronegative patients (*p* > 0.05). The seroprevalence rate was lower-than-expected among Kuwaiti patients (2/307, 0.7%) in comparison with non-Kuwaiti patients (8/57, 14.0%) (χ2 = 33.603, *df* = 1, *p* < 0.001) who originated from endemic South/Southeast Asian countries. Seven out of 8 (87.5%) seropositive patients had a history of direct contact with cats, dogs, or both. The seroprevalence rate of toxocariasis among allergic patients in Kuwait was 2.5%. Raising awareness and early deworming treatment/prophylaxis for juvenile dogs and cats remain crucial for toxocariasis prevention.

## 1 Introduction

Toxocariasis is a worldwide helminthic infection caused mainly by the dog roundworm *Toxocara canis*, and less commonly by the cat roundworm *Toxocara cati*. Infected dogs and cats excrete parasite eggs into the environment during defecation, after which humans acquire the infection through accidental ingestion of embryonated parasite eggs. After ingestion, *Toxocara* eggs hatch and the larvae penetrate the intestinal wall into the blood stream and migrate to different parts of the body, including the eyes, brain, heart, liver and lungs. In these organs, the larvae cause local inflammation which leads to non-specific symptoms. Visceral larva migrans may manifest as encephalitis, arrhythmia and hypoxia, while ocular larva migrans commonly manifests as blurred vision, floaters, photophobia, leukocoria, and/or ocular inflammation. Due to the severity of the illness, number of infected individuals, and availability of treatment, toxocariasis is placed among the top 6 parasitic diseases in the USA which are prioritized for public health action (“CDC - Parasites - Neglected Parasitic Infections (Npis) in the United States.”, 2020).

Peripheral blood eosinophilia (> 500 eosinophils/μl) (Kuang, 2020) may be associated with a long list of medical conditions which include parasitic infections, allergic rhinitis, asthma, eosinophilic pneumonia, drug-induced allergies, connective tissue and autoimmune diseases, blood-related and solid organ-associated malignant neoplasms, and solid organ transplant rejection (Kovalszki and Weller, 2016). Toxocariasis infection is associated with the activation and upregulation of eosinophils in the peripheral blood, spleen, and bone marrow, enhanced connectivity with T-cells and increased expression of CD69/MHC-II/CD80/CD86 genes (Rodolpho et al., 2018). A systematic review and meta-analysis which included 10 studies and >1500 subjects had reported a significantly higher prevalence of *T. canis* infection in patients with asthma than in controls (*p* < 0.001) (Li et al., 2014). The association of *Toxocara* seropositivity with eosinophilia has also been documented in multiple studies which reported that 22–65% of patients with idiopathic eosinophilia had anti-*Toxocara* IgG antibodies (Incorvaia et al., 2011; Kim et al., 2017; Qualizza et al., 2011; Song et al., 2020; Yoon et al., 2018). Furthermore, a previous study reported 3 cases which were initially misdiagnosed with allergies (dermatitis, rhinitis and asthma) and were later found to have toxocariasis infection via serological testing (Qualizza et al., 2009).

The prevalence of toxocariasis varies widely by geography and climate, ranging from 1.4% among nomads in Iran (Arefkhah et al., 2019) to 92.4% among pregnant women in Nigeria (Ikotun et al., 2020). Humans and cats have co-existed for many decades in Kuwait, where stray cats are commonly seen scavenging for food scraps in rubbish bins near residences. On the other hand, stray dogs were rarely seen previously but are becoming a more common phenomenon due to the increased importation of exotic dog breeds and owner abandonment. There has been a rising trend in pet dog and cat ownership in Kuwait during the past decade with a corresponding increase in the number of imported dog and cat breeds, commercial establishments which include >10 privately owned veterinary hospitals and clinics, and an abundance in pet supply stores, grooming salons, pet hotels and cafes, and adoption centers (Kuwait News Agency, 2018). To our knowledge, there are no reports on human toxocariasis in the State of Kuwait or in the other Gulf Cooperation Council (GCC) countries of the Arabian Peninsula. Therefore, we investigated the seroprevalence of toxocariasis among allergic patients in Kuwait and examined the association of *Toxocara* seroprevalence with eosinophilia and other factors including age, gender, nationality, and history of direct contact with dogs or cats.

## 2 Materials and methods

### 2.1 Selection of patient data and specimens

During the period from September to December 2021, the laboratory records of allergic patients referred from primary healthcare centers to Al-Rashed Allergy Hospital, Kuwait were reviewed. The selected study groups included allergic patients from both genders and all ages and nationalities. The sole criterion for the selection of samples was the peripheral blood eosinophil count reviewed from recent complete blood count (CBC) results. Accordingly, a total of 400 serum samples were selected: 200 samples from patients with normal eosinophil count (< 500 cells/μl) and 200 samples from patients with eosinophilia (> 500 cells/μl).

### 2.2 Collection of specimens and screening of anti-*Toxocara* IgG antibodies

After the selection of specimens, 0.5 ml of serum was collected from pre-collected, stored sera at 5 °C in the Hematology Laboratory, Al-Rashed Allergy Hospital. The collected sera were transported to the Research Core Facility (RCF), Faculty of Medicine, Kuwait University, and stored at −20 °C until further use. The sera were screened for the presence of anti-*Toxocara* IgG antibodies using an antibody enzyme-linked immunosorbent assay (Ab-ELISA) kit (TECAN® - IBL International GmbH, Hamburg, Germany) with 96.9% sensitivity and 98.6% specificity according to the manufacturer's instructions. All seropositive samples were additionally screened for anti-*Ascaris lumbricoides* IgG and anti-*Schistosoma mansoni* IgG antibodies using Ab-ELISA kits (TECAN® - IBL International GmbH) to rule out possible cross-reactivity.

### 2.3 Collection of clinical history from seropositive patients

The *Toxocara*-seropositive patients were asked about their history of direct contact with dogs or cats and the responses were recorded. Additionally, the seropositive patients with eosinophilia were asked a series of questions to rule out other confounding factors for eosinophilia (i.e., history of other parasitic infections, asthma, itchy rash, autoimmune diseases, malignancies, and intake of antibiotics or herbal supplements).

### 2.4 Statistical analysis

Descriptive and in-depth statistical analyses were performed using the Microsoft Excel® Analysis ToolPak software. The overall seroprevalence rate of toxocariasis among the allergic patients and the percentage of seropositive patients who had a history of direct contact with dogs and/or cats were calculated. The Student's *t*-test was used to compare the mean age of the *Toxocara*-seropositive patients with that of the seronegative patients. The Chi-square and linear regression tests were applied to examine the association of *Toxocara* seropositivity with eosinophilia, age, gender, and nationality. A *p* value <0.05 was considered as statistically significant.

## 3 Results

Out of 400 referred allergic patients, only 10 (2.5%) had anti-*Toxocara* IgG antibodies. Six out of 10 (60%) seropositive patients were female and 4 (40%) were male. Five seropositive patients had a normal eosinophil count (< 500 cells/μl) while 5 had peripheral eosinophilia (> 500 cells/μl). Four out of 10 (40%) seropositive patients originated from the Philippines, 3 (30%) from Sri Lanka, 2 (20%) from Kuwait and 1 (10%) from Bangladesh.

The age of allergic patients (*n* = 400) ranged from 2 to 85 years with a mean age of 35.6 ± 1.8 years (95% CI: 33.8–37.4). There was no difference in mean age between *Toxocara*-seropositive patients [38.7 ± 8.8 years (95% CI: 29.9–47.5)] and seronegative patients [35.4 ± 1.8 years (95% CI: 33.6–37.2)] (*p* = 0.431). There was no association between age and *Toxocara* seropositivity [R^2^ = 0.000, F(1, 398) = 0.037, *p* = 0.848)].

Most of the allergic patients were female (238/400, 59.5%) while 162/400 (40.5%) were male. By gender, there was no difference in *Toxocara* seropositivity between male (4/162, 2.5%) and female (6/238, 2.5%) allergic patients (χ^2^ = 0.051, *df* = 1, *p* = 0.974). However, the number of male allergic patients with eosinophilia (94/162, 58.0%) was higher-than-expected in comparison with female patients with eosinophilia (106/238, 44.5%) (χ^2^ *=* 4.943, *df* = 1, *p* = 0.008).

Out of 400 allergic patients, the nationalities of 361 patients were known, and originated from 17 countries as follows: Kuwait (307/361, 85.0%), Saudi Arabia (11/361, 3.0%), Egypt (9/361, 2.5%), the Philippines (6/361, 1.7%), Bangladesh (5/361, 1.4%), India and Sri Lanka (4/361 each, 1.1%), Iraq (3/361, 0.8%), Lebanon, Pakistan and Yemen (2/361 each, 0.6%), and Afghanistan, Ethiopia, Jordan, Oman, Sudan, Syria (1/361 each, 0.3%). The *Toxocara*-seropositive allergic patients (*n* = 10) originated from 4 countries as follows: the Philippines (4/10, 40.0%), Sri Lanka (3/10, 30.0%), Kuwait (2/10, 20.0%), and Bangladesh (1/10, 10.0%). Although the large majority of allergic patients were Kuwaiti nationals, *Toxocara* seropositivity was lower-than-expected among Kuwaiti patients (2/307, 0.7%), but higher-than-expected among non-Kuwaiti patients (8/57; 14.0%) (χ2 = 33.603, *df* = 1, *p* < 0.001) who originated from South/Southeast Asian countries, particularly Sri Lanka (3/4, 75.0%), the Philippines (4/6, 66.7%), and Bangladesh (1/5, 20.0%) (χ2 = 159.638, *df* = 3, *p* < 0.001). Table 1 summarizes the demographic data and *Toxocara* seropositivity among the allergic patients.

**Table 1:** Seroprevalence rate of toxocariasis among allergic patients in Kuwait by age, gender and nationality.

Variable	Total (*n* = 400)	Seronegative (*n* = 390)	Seropositive (*n* = 10)	*p* value	
**Age** mean (years)	35.6 ± 1.8 (95% CI: 33.8–37.4)	35.4 ± 1.8 (95% CI: 33.6–37.2)	38.7 ± 8.8 (95% CI: 29.9–47.5)	0.431	
**Gender**				0.974	
Male	162 (40.5%)	158 (40.5%)	4 (40.0%)		
Female	238 (59.5%)	232 (59.5%)	6 (60.0%)		
**Nationality**^1^				< 0.001	
Kuwait	307 (85.0%)	305 (84.5%)	2 (20.0%)		
Philippines	6 (1.7%)	2 (0.6%)	4 (40.0%)		
Sri Lanka	4 (1.1%)	1 (0.3%)	3 (30.0%)		
Bangladesh	5 (1.4%)	4 (1.1%)	1 (10.0%)		
Others 2	39 (10.8%)	39 (10.8%)	0		

1 Nationality: Percentage calculation is based on the total number of patients of known nationality (*n* = 361).

2 Others: Saudi Arabia (11/361, 3.0%), Egypt (9/361, 2.5%), India (4/361, 1.1%), Iraq (3/361, 0.8%), Lebanon, Pakistan, Yemen (2/361 each, 0.6%), and Afghanistan, Ethiopia, Jordan, Oman, Sudan, Syria (1/361 each, 0.3%).

Out of 10 seropositive patients, only 8 were available for the collection of clinical history (4 with eosinophilia and 4 with normal eosinophil count). Six out of 8 patients (75.0%) were referred for symptoms of urticaria, while 2 (25.0%) were referred for symptoms of asthma. Two patients were referred due to symptoms of systemic rash/urticaria after administering COVID-19 vaccination. The confounding factors for eosinophilia in 4 seropositive patients were as follows: Patient 1 (urticaria caused by COVID-19 vaccination); Patient 2 (urticaria due to shellfish allergy); Patient 3 (asthma + antibiotic use of amoxicillin/clavulanic acid); Patient 4 (idiopathic urticaria). Seven out of 8 (87.5%), 5 out of 8 (62.5%) and 4 out of 8 (50.0%) seropositive allergic patients had a history of direct contact with cats, dogs, or both, respectively. Four out of 7 (57.1%) patients had kittens or juvenile cats <1 year of age. Three (37.5%) seropositive patients had direct contact with cats and/or dogs only in their country of origin (2 from Sri Lanka, 1 from the Philippines).

Four out of 10 (40%) *Toxocara*-seropositive patients had anti-*Ascaris lumbricoides* IgG antibodies, while 2 out of 10 (20%) had anti-*Schistosoma mansoni* IgG antibodies. However, all 10 *Toxocara*-seropositive patients showed higher ELISA units against *T. canis* in comparison with *A. lumbricoides* and *S. mansoni* (Table 2). Two *Toxocara*-seropositive female patients from the Philippines with normal eosinophil count were seropositive for both *A. lumbricoides* and *S. mansoni*, while 1 female patient from the Philippines with eosinophilia had IgG antibodies against *A. lumbricoides* alone. One female patient from Sri Lanka with normal eosinophil count had anti-*A. lumbricoides* IgG antibodies.

**Table 2:** Demographic data of Toxocara-seropositive allergic patients and IgG cross-reactivity with Ascaris lumbricoides and *Schistosoma mansoni* antigens.

Patient ID 1	Age	Gender	Nationality	ELISA Units (U); Cut-off = 10 U; Positive >11 U		
				*Toxocara canis*	*Ascaris lumbricoides*	***Schistosoma mansoni***
N119	29	F	Philippines	50.23	13.55	25.23
N126	46	M	Bangladesh	11.74	4.23	5.13
N134	52	F	Sri Lanka	16.62	14.00	8.74
N137	39	F	Philippines	15.31	11.76	12.11
N157	20	M	Kuwait	17.55	2.96	3.93
E48	32	M	Sri Lanka	16.95	6.32	5.88
E49	62	M	Kuwait	14.21	2.91	7.90
E50	41	F	Sri Lanka	16.42	9.70	5.35
E152	30	F	Philippines	43.45	5.13	6.32
E188	36	F	Philippines	31.41	17.59	4.41

1 ‘N' represents patient with normal eosinophil count; ‘E' represents patient with eosinophilia.

## 4 Discussion

Globally, the seroprevalence of toxocariasis varies widely by climate and geographical region, being generally lower in industrialized temperate countries such as the USA (3.6%) (Bradbury et al., 2020) and China (5.1%) (Wang et al., 2020), and higher in lower-middle-income tropical countries such as Nigeria (92.4%) (Ikotun et al., 2020), Honduras (88.6%) (Hernández et al., 2020), Ghana (62.0%) (Boyko et al., 2020), and Vietnam (59.0%) (Van De et al., 2020). Our study reported a relatively low toxocariasis seroprevalence rate of 2.5% among allergic patients, 80% of whom were non-Kuwaiti residents originating from lower-middle-income South/Southeast Asian tropical countries (i.e., the Philippines, Sri Lanka and Bangladesh). This finding corresponds to the population of non-Arab Asian immigrants which constitutes one-third of the total population (~ 4.4 million persons) in Kuwait (Kuwait Population, 2022).

Several studies have reported a high prevalence of soil-transmitted helminths in South/Southeast Asia. A study from Laguna, Philippines showed that 460 out of 1480 (31%) soil samples were contaminated with soil-transmitted helminths with a remarkable prevalence of *Toxocara* spp. (77%) in comparison with other helminths [*Ascaris* spp. (11%); hookworms/strongyles/free-living nematodes (7%); *Trichuris* spp. (5%)] (Paller et al., 2014). Another study also reported a high rate (43%) of soil contamination with *Toxocara* eggs at a concentration of 1 egg/g of soil, which was positively correlated with the *Toxocara* seroprevalence among school children (49%) (Fajutag, 2013). A considerable prevalence of canine toxocariasis has been reported in Cebu, Philippines, where a study detected *T. canis* eggs in 23 out of 200 (11.5%) owned and sheltered dogs (Urgel et al., 2019). A recent study from Sri Lanka reported that *Toxocara* eggs were found in 4.5% of collected soil samples and 17% of fecal samples collected from stray dogs (Galgamuwa et al., 2020). The seroprevalence rate of human toxocariasis was even higher among suspected cases of visceral larva migrans (50%) and ocular toxocariasis (62%), particularly in children below the age of 14 years (Iddawela et al., 2017a; Iddawela et al., 2017b).

The prevalence of *Toxocara* eggs has been strongly correlated with the moisture content of soil (*r* = 0.799; *p* = 0.027) (Paller et al., 2014). A study from Iran reported a strong correlation between the prevalence of *T. canis* excretory-secretory (TCES)-specific IgG antibodies and contact with soil (*p* < 0.001), dogs (*p* < 0.001), and residing in a village (*p* = 0.006) (Ashtari et al., 2019). Another study reported higher prevalence rates of *T. cati* (28.5%) and *T. canis* (13.8%) in humans in the northern regions of the Iran due to optimum climate conditions and close contact between humans and host animals (Eslahi et al., 2020). The State of Kuwait has an arid environment characterized by very hot and dry weather during the long summer months (May–September) when temperatures commonly exceed 50 °C, particularly from June to August, and scarce rainfall during the winter months (November–February). Thus, it is highly probable that the climate of Kuwait creates a stressful environment which inhibits the survival of soil-transmitted helminths including *Toxocara* eggs, and consequently breaks a major route of transmission. Moreover, the extreme temperatures during the long summer months in Kuwait drastically limit the outdoor recreational activities and thus reduce the frequency of human contact with soil. Nearly all (>99.9%) Kuwaiti nationals are Muslims who are religiously prohibited from keeping dogs as pets inside their homes since dog saliva is deemed to be an impurity, but dogs may be employed for outdoor activities such as hunting, guarding and animal herding. These factors may explain the low seroprevalence rate of toxocariasis, especially among Kuwaiti nationals (0.7%). The veterinary hospitals in Kuwait routinely prescribe antihelminthic treatment/prophylaxis (pyrantel pamoate/oxantel pamoate) to deworm dogs and cats starting from 6 weeks of age and every 6 months thereafter. A recent study from Grenada reported that 147 out of 306 (48%) puppies and juvenile dogs below the age of 1 year were infected with *T. canis*, while puppies aged as young as 2 to 12 weeks were the highest shedders of *T. canis* eggs (Schwartz et al., 2022). Furthermore, *Toxocara* infection may be present in 51–100% of puppies and up to 91% of juvenile cats (Rostami et al., 2019). Accordingly, it is recommended that puppies are initially treated at 2, 4, 6 and 8 weeks of age and kittens at 3, 5, 7 and 9 weeks of age, followed by monthly treatment until 6 months of age, and then annually, biannually, or tri-annually thereafter (Macpherson, 2013). Hence, it is necessary to administer antihelminthic treatment/prophylaxis at an earlier age for dogs (2 weeks) and cats (3 weeks) in veterinary hospitals and clinics in Kuwait. Our study showed that the majority of *Toxocara*-seropositive allergic patients had a history of direct contact with cats and/or dogs, which is the probable source of local *Toxocara* transmission in Kuwait.

The association between eosinophilia and *Toxocara* seropositivity has been well-established in previous studies from highly endemic countries such as Brazil, Ecuador and Peru, particularly among the hyper-eosinophilic patients (>10%) (Dattoli et al., 2011; Morales-Yánez et al., 2020; Roldán et al., 2008). However, the low number of seropositive patients (*n* = 10) reported in our study and the equal distribution of *Toxocara* seropositivity among eosinophilic (*n* = 5) and non-eosinophilic (*n* = 5) allergic patients rendered it difficult to verify the association between eosinophilia and *Toxocara* seropositivity. Nonetheless, circulating anti-*Toxocara* IgG antibodies can persist for >3 years (Elefant et al., 2006), long after the resolution of infection and normalization of eosinophil levels. Therefore, a positive ELISA result should not be interpreted as a confirmation of active toxocariasis. Furthermore, our study showed that 4 *Toxocara*-seropositive patients had anti-*Ascaris lumbricoides* IgG antibodies and 2 *Toxocara*-seropositive patients had anti-*Schistosoma mansoni* IgG antibodies. This finding makes it difficult to determine whether the *Toxocara*-specific IgG antibodies from patients had an affinity to similar antigens from other parasite species (cross-reactivity), or if a patient was previously infected with multiple parasite species. Therefore, the development of DNA-based methods to confirm active toxocariasis would be of higher diagnostic value than the current gold standard (ELISA).

Similar to previous studies, we have not found an association between *Toxocara* seropositivity and the age (Dattoli et al., 2011; Khozime et al., 2018) or gender (Ashtari et al., 2019; Khozime et al., 2018) of the allergic patients. However, two studies from South Korea had reported higher *Toxocara* seropositivity among male patients (Song et al., 2020; Yoon et al., 2018), particularly those with a history of consuming the raw meat/livers of animals and heavy alcohol consumption (Song et al., 2020).

## 5 Conclusion

The overall seroprevalence rate of toxocariasis among allergic patients in Kuwait was 2.5%. Seropositivity was higher among non-Kuwaiti patients who originated from South/Southeast Asian countries and had a history of direct contact with cats, dogs, or both. Hence, it is important to raise awareness and perform early deworming treatment/prophylaxis for puppies (2-week-old) and kittens (3-week-old), and to screen human patients with idiopathic allergies for anti-*Toxocara* IgG antibodies.

## Ethics approval

Ethical approval to carry out this research was obtained from the Ethical Committee of the Ministry of Health [Project Number 1759/2021], Kuwait. All subjects involved in this study remain anonymous and all collected data are strictly confidential and used solely for statistical purposes.

## Declaration of Competing Interest

The authors declare that they have no conflict of interest.
